# Evaluation of an artificial intelligence clinical trial matching system in Australian lung cancer patients

**DOI:** 10.1093/jamiaopen/ooaa002

**Published:** 2020-05-01

**Authors:** Marliese Alexander, Benjamin Solomon, David L Ball, Mimi Sheerin, Irene Dankwa-Mullan, Anita M Preininger, Gretchen Purcell Jackson, Dishan M Herath

**Affiliations:** o1 Department of Pharmacy, Peter MacCallum Cancer Centre, Melbourne, Victoria, Australia; o2 Sir Peter MacCallum Department of Oncology, University of Melbourne, Parkville, Victoria, Australia; o3 Department of Medical Oncology, Peter MacCallum Cancer Centre, Melbourne, Victoria, Australia; o4 Department of Radiation Oncology, Peter MacCallum Cancer Centre, Melbourne, Victoria, Australia; o5 IBM Watson Health, Cambridge, Massachusetts, USA

**Keywords:** clinical trial matching, machine learning, natural language processing

## Abstract

**Objective:**

The objective of this technical study was to evaluate the performance of an artificial intelligence (AI)-based system for clinical trials matching for a cohort of lung cancer patients in an Australian cancer hospital.

**Methods:**

A lung cancer cohort was derived from clinical data from patients attending an Australian cancer hospital. Ten phases I–III clinical trials registered on clinicaltrials.gov and open to lung cancer patients at this institution were utilized for assessments. The trial matching system performance was compared to a gold standard established by clinician consensus for trial eligibility.

**Results:**

The study included 102 lung cancer patients. The trial matching system evaluated 7252 patient attributes (per patient median 74, range 53–100) against 11 467 individual trial eligibility criteria (per trial median 597, range 243–4132). Median time for the system to run a query and return results was 15.5 s (range 7.2–37.8). In establishing the gold standard, clinician interrater agreement was high (Cohen’s kappa 0.70–1.00). On a per-patient basis, the performance of the trial matching system for eligibility was as follows: accuracy, 91.6%; recall (sensitivity), 83.3%; precision (positive predictive value), 76.5%; negative predictive value, 95.7%; and specificity, 93.8%.

**Discussion and Conclusion:**

The AI-based clinical trial matching system allows efficient and reliable screening of cancer patients for clinical trials with 95.7% accuracy for exclusion and 91.6% accuracy for overall eligibility assessment; however, clinician input and oversight are still required. The automated system demonstrates promise as a clinical decision support tool to prescreen a large patient cohort to identify subjects suitable for further assessment.

## BACKGROUND AND SIGNIFICANCE

Prospective clinical trials are the gold standard for assessing the potential harms and benefits of new cancer treatments. However, clinical trial recruitment is challenging and time-consuming.^1^^,^^2^ As of 2018, only about 6% of patients with a cancer diagnosis in the state of Victoria in Australia were recruited to clinical trials, with rates unchanged for more than a decade.

While the global increase in the availability of clinical trials should facilitate greater trial recruitment, a key deterrent is the tedious manual task of matching patients to clinical trials or identifying a cohort of patients for a trial. Both processes require detailed knowledge of patient characteristics and trial eligibility criteria, a challenge given the growing number clinical trials available and complexity of trial designs. Eligibility criteria for clinical trials can narrowly define the study population and thus limit the number of patients who can enroll in clinical trials. The time to screen patients for trials can further limit enrollment.

Studies demonstrate that incorporating clinical decision support into the management of oncology patients and automating referrals to clinical trials show promise for increased patient referrals.^1^^,^^3–9^ IBM^®^ (Cambridge, MA, United States) Watson for clinical trial matching (CTM) is a software platform developed to identify potential trials for individual patients or potential trial candidates for individual trials. CTM uses natural language processing (NLP) to ingest trial and patient information from unstructured sources and matches patients to trials for which they might be eligible through machine learning (ML) techniques. Previous studies have shown that CTM^10^ can reduce the screening time for clinical trials and increase trial enrollment.^3^

CTM determines the degree of eligibility based on the patient clinical attributes entered. Features of the tool include the ability to classify patients as “Exclude” (patient not eligible) or “Consider” (patient potentially eligible), based on patient attributes and whether the patient has unmet criteria which are modifiable conditions. This mimics real-world practice and circumstances faced by clinical trial screeners in which individual records are examined successively against increasingly specific criteria to identify modifications that can increase a patient’s chance for matching to a trial.

The objective of this retrospective study was to evaluate the performance of CTM eligibility determinations against a selected pool of active lung cancer trials for a cohort of potentially eligible patients in an Australian cancer hospital.

## METHODS

### Participants

The lung cancer cohort was derived from a clinical dataset of patients attending an Australian specialist cancer hospital and enrolled a prospective observational cohort study—the Thoracic Malignancies Cohort (TMC). The hospital institutional review board (IRB) approved the TMC (study no. 17/70, approved January 4, 2012) as a prospective observational study; consenting patients are followed from diagnosis or first hospital presentation at 3-month intervals until death or loss to follow-up. The IRB approval and patient consent allow the TMC data to be used in other IRB approved studies, including the current study (study no. 17/152L, approved October 24, 2017).

Eligibility criteria for this study included diagnosis of small cell lung cancer (SCLC) or non-small cell lung cancer (NSCLC) between 2012 and 2018. A pragmatic sample of approximately 100 cases was selected based on most recent study follow-up, in reverse chronological order from September 1, 2018. This data extraction date was selected to allow at least 3 months for study follow-up at time of data extraction (December 1, 2018), ensuring at least 1 routine follow-up and complete data acquisition. Follow-up date, rather than diagnosis or first presentation date, identified cases that defined a representative sample of patients attending lung oncology clinics during the study period. These selection criteria represent an unselected patient cohort who may be considered for a range of clinical trials including all stages of disease and clinical time points within a patient’s journey, including newly diagnosed patients, treatment-naïve patients, previously treated patients, and patients receiving ongoing treatment.

### Data collection

Clinical trial eligibility criteria were extracted for 10 phases I–III cancer clinical trials registered on clinicaltrials.gov that were open to lung cancer patients at the Peter MacCallum Cancer Center in Melbourne, Australia. Because inclusion and exclusion criteria in ClinicalTrials.gov often require clarification, additional detail required for the CTM protocol ingestion was obtained from relevant trial protocols. For example, an exclusion criterion in ClinicalTrials.gov may state “no therapy allowed,” however, the institution may clarify this by stating “no previous chemotherapy or radiotherapy allowed” in the institutional protocol. CTM allows users to ingest eligibility criteria from ClinicalTrials.gov and modify as needed. Watson’s NLP algorithms extracted eligibility and exclusion criteria from the protocol library of Portable Document Format files that contained previously extracted inclusion and exclusion criteria for trials. The trial data intake was optimized with 3 rounds of trial ingestion and evaluated by experts to validate ingestion protocols prior to study inception.

Clinical data for included patients were extracted from the TMC study database and medical records. De-identified patient attributes such as histological diagnosis, stage, and prior therapies were manually entered in CTM. The TMC database collects the following variables at diagnosis: TNM staging according to 7th and 8th edition of UICC staging criteria (as relevant for year of cancer diagnosis), histological subtype (adenocarcinoma, squamous cell carcinoma, large cell carcinoma, NSCLC not otherwise specified, carcinoid, SCLC), mutation status (epidermal growth factor receptor [EGFR], anaplastic lymphoma kinase [ALK], Kirsten rat sarcoma viral oncogene homolog [KRAS], BRAF, MET); PDL1 expression; comorbidities according to the Simplified Comorbidity Score^11^ including tobacco consumption, diabetes mellitus, renal insufficiency, respiratory comorbidity, cardiovascular comorbidity, neoplastic comorbidity, and alcoholism; Eastern Cooperative Oncology Group performance status (PS); weight loss within 3 months of diagnosis (0–10%, 11–15%, >15%); smoking history (current, past, never); smoking magnitude (pack-years); sex, and age. Longitudinal data include cancer treatment (chemotherapy, immunotherapy, targeted therapy, radiotherapy, surgery), patient status, and response to therapy. Results of specific diagnostic tests not mandated by the TMC study are collected and reported if testing is performed as part of routine care.

CTM collects the following attributes: primary cancer staging, metastatic disease (bone, brain, liver, lung CNS, epidural, meningeal, leptomeningeal), PS, mutations (ALK, EGFR, BRAF, ERBB2, MET, NTRK1–3, PDL1, RAS [KRAS, NRAS, HRAS], RET, ROS), prior cancer therapy (chemotherapy in any setting, adjuvant/neoadjuvant setting or metastatic setting, platinum chemotherapy, hormone therapy, radiotherapy [radiosurgery, whole brain radiotherapy]), lung surgery including surgical type, cancer histology, demographics (age, gender, race), echocardiography, pathology (FBE, U&E, other), past medical history, medications, comorbidities, current setting (metastatic, adjuvant, neoadjuvant), and current status (progressive disease).

### Statistical analysis

This study tested the overall performance (including ML and NLP) of the CTM eligibility determination process. CTM-processed clinical trial eligibility criteria were checked and refined by 2 clinicians (a medical oncologist and a pharmacist) prior to commencement of matching. Accuracy of NLP processing of eligibility criteria from trial protocol extracts was not evaluated, because the eligibility criteria in ClinicalTrials.gov was modified to include additional protocol details, including laboratory results not included in the database. Once matching was complete, a timed query was executed using a cloud-based instance of CTM. Each patient was assessed for eligibility against potential trials and classified by CTM as “Exclude” (patient not eligible) or “Consider” (patient potentially eligible).

A gold standard for trial eligibility was determined for each patient and the 10 cancer trials by manual review of patient attributes entered into CTM (not the full medical record) by 2 clinicians, with discrepancies discussed to achieve consensus. Accuracy (agreement), recall (sensitivity), specificity, and precision (positive predictive value [PPV] and negative predictive value [NPV]) of CTM trial classification was measured against this gold standard. CTM performance was further classified by counts (per trial and overall) of the total number of individual inclusion/exclusion criteria assessed by CTM and the proportion of assessments that agreed with the gold standard. Interrater reliability between clinicians involved in manual review leading to a consensus gold standard was measured by Cohen’s kappa with reported standard error.

## RESULTS

**Figure 1.**
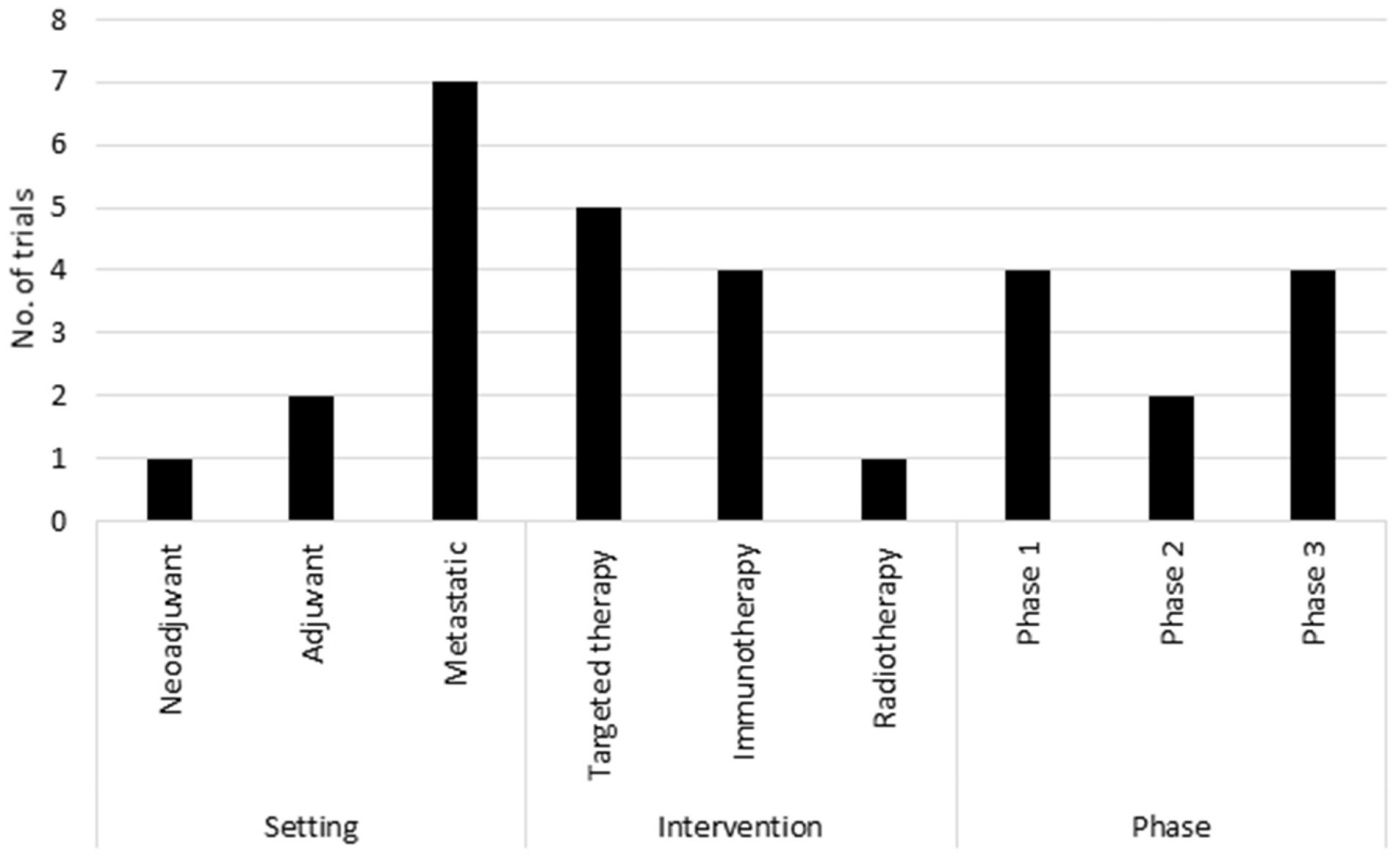
Clinical trial setting, intervention, and phase. Summary of patient attributes and clinical trial features.

A total of 102 lung cancer patients were included in the study and assessed for eligibility against 10 lung cancer clinical trials. Patient attributes and trial features are summarized in Table 1 and Figure 1, respectively. More detailed trial descriptions are available in Supplementary Table S1.

**Table 1.** Selected patient attributes entered in CTM

Demographics	No.	%
Age, median (range)	69 (26–91)	
Sex, male	57	56
Race		
Caucasian	40	39
Asian	11	11
Other	2	2
Not stated^a^	49	48
Cancer staging		
Metastatic at diagnosis	42	41
Metastatic at screening	46	45
Measurable disease		
Yes	36	35
No	36	35
Unknown/requires assessment	30	29
Histology and mutation		
NSCLC, non-squamous	71	78
NSCLC, squamous	20	28
SCLC	4	4
Neuroendocrine	2	2
No histologic diagnosis	5	5
Mutation positive		
EGFR	21	21
ALK	11	11
KRAS	5	5
BRAF	2	2
ROS1	0	0
PDL1 expression known and >1%	10	10%
Prior cancer treatment		
Any chemotherapy	57	56
Neoadjuvant or adjuvant chemotherapy	9	9
Metastatic chemotherapy	30	29
Platinum chemotherapy	30	29
Biologic/targeted therapy	26	25
Immunotherapy	20	20
Any radiotherapy	82	80
Any lung surgery	22	22
Other		
ECOG performance status, 0–1	71	70
ECOG performance status, ≥2	28	27
ECOG unknown	4	2
History prior malignancy	22	22
Current smoker	12	12

^a^ *Note:* Attributes presented as entered into CTM with all field accurately representing data from the prospective clinical database expect for race which was available for all patients, however, only entered in CTM for a portion of patients due to data entry omission.

*Abbreviations:* ALK: anaplastic lymphoma kinase; BRAF: proto-oncogene B-Raf; CTM: clinical trial matching; ECOG: Eastern Cooperative Oncology Group; EGFR: epidermal growth factor receptor; KRAS: Kirsten rat sarcoma viral oncogene homolog; NSCLC: non-small cell lung cancer; PDL1: programmed death ligand 1; ROS1: ROS proto-oncogene 1; SCLC: small cell lung cancer.

CTM evaluated a total of 7252 patient attributes (per patient median 74, range 53–100) against 11 467 individual trial eligibility criteria (per trial median 597, range 243–4132). This dataset consisted of 4818 patient attributes entered by clinicians (per patient median 49, range 24–78) and 2434 attributes derived by CTM (per patient median derivation of an additional 25 attributes by CTM, range 16–33).

The median time for CTM to run a query and return eligibility determinations for 102 patients against 10 trials was 15.5 s (range 7.2–37.8 s). In establishing the gold standard comparator, clinician interrater agreement was high (Cohen’s kappa 0.70–1.00, Table 2), with disagreement due to selection error or overlooked features/criteria, rather than material disagreement. Agreement was lowest for trial 10, in which clinician interpretation of exclusion criteria relating to “relevant” driver mutations was discrepant. Differences were reconciled on discussion, which determined that patients with driver mutations would remain potentially eligible, due to the lack of detail provided for classification of excluded mutations. This detail was included only in trial appendices and thus not available to CTM for processing, nor considered in clinician assessments. Excluding this trial, agreement levels were ≥97% (Cohen’s kappa 0.82–1.00). On a per-patient basis, the accuracy of CTM for eligibility classification across trials was 91.6%; recall (sensitivity), 83.3%; precision, (PPV) 76.5%; NPV, 95.7%; and specificity was 93.8%. When considering trials classified as “Exclude” by CTM, accuracy was 95.7%, with 34 of 799 trials incorrectly excluded. Conversely, for trials classified as “Consider” by CTM, accuracy was 76.5%, with 52 of 221 labeled “Consider” that should have been excluded. CTM accuracy for individual trials ranged from 77% to 100% (Table 2).

**Table 2.** Clinician and CTM agreement with gold standard for overall trial eligibility status

Trial ID	Number of criteria assessed	Clinician interrater agreement for establishing gold standard			% patients listed for trial consideration		CTM and gold standard
		Agreement (%)	Kappa	Std. Err	CTM (%)	Gold standard (%)	Agreement (%)
1	270	100	1.000	0.099	1	2	99
2	560	97	0.872	0.098	1	1	92
3	243	100	NR	NR	0	0	100
4	1010	99	0.967	0.099	23	18	93
5	313	98	0.823	0.099	3	6	97
6	4132	99	0.980	0.099	59	40	77
7	634	97	0.919	0.099	11	25	78
8	486	100	1.000	0.099	8	5	97
9	1795	100	1.000	0.099	49	48	93
10	2024	85	0.703	0.096	53	46	89

*Abbreviations:* CTM: clinical trial matching; NR: not reported; Std. Err: standard error.

Among all trials, 1490 trial eligibility criteria (inclusion/exclusion) were listed as “not met” by CTM (90% agreement with gold standard), 1231 were “met” (96% agreement), 8088 were identified as requiring further action to make a decision and listed as “action needed” (89% agreement), 136 were identified as “unmet modifiable” (90% agreement), and 522 consent criteria were reviewed (81% agreement). The number of data elements and criteria varied by trial, with trial 6 representing an umbrella multicohort design with a notably higher number of criteria. Agreement between CTM and gold standard was lower for trials 6 and 7, reflecting complexity of trial 6 and interpretation difficulties by CTM relating to technicalities of exclusion for prior radiotherapy in trial 7. For each clinical trial, the total number of items assessed, their CTM classifications, and accuracy are detailed in Table 3.

**Table 3.** CTM assessment of individual trial inclusion and exclusion criteria

Trial ID	Median (range), per pt	Total	Agreement		Median (range), per pt	Total	Agreement		Median (range), per pt	Total	Agreement		Median (range), per pt	Total	Agreement	
			No.	%			No.	%			No.	%			No.	%
	Not met inclusion criteria				Not met exclusion criteria				Met inclusion criteria				Met exclusion criteria			
1	2 (0–4)	236	233	99	0 (0–2)	4	3	75	4 (4–4)	4	4	100	2 (2–2)	2	2	100
2	1 (0–4)	143	138	97	0	0	0	–	5 (3–8)	58	58	100	0	0	0	–
3	3 (1–4)	243	237	98	0	0	0	–	0	0	0	–	0	0	0	–
4	1 (0–4)	130	126	97	0	0	0	–	5 (3–6)	105	104	99	5 (1–7)	106	105	99
5	1 (0–3)	135	124	92	0	0	0	–	7 (4–9)	27	27	100	1 (0–1)	2	2	100
6	1 (0–2)	79	65	82	0 (0–2)	28	28	100	5 (2–6)	229	218	95	3 (0–7)	138	106	77
7	1 (0–3)	93	63	68	0 (0–1)	18	1	6	5 (3–7)	68	68	100	2 (0–4)	26	26	100
8	2 (0–5)	253	198	78	0	0	0	–	5 (5–6)	32	31	97	1 (1–1)	6	6	100
9	1 (0–2)	56	56	106	0	0	0	–	3 (1–5)	124	124	100	0	0	0	–
10	0 (0–3)	72	68	94	0	0	0	–	5 (2–8)	253	251	99	1 (0–2)	51	49	96
All trials		1440	1308	91		50	32	64		900	900	885		331	296	89
	Action needed inclusion criteria				Action needed exclusion criteria				Unmet modifiable condition				Consent criteria			
1	8 (8–8)	8	7	88	13 (13–13)	13	11	85	0	0	0	–	3 (3–3)	3	2	67
2	7 (5–10)	82	75	91	23 (22–23)	250	232	93	1 (0–1)	7	7	100	2 (1–2)	20	12	60
3	0	0	0	–	0	0	0	–	0	0	0	–	0	0	0	–
4	2 (1–3)	47	43	91	23 (22–29)	531	484	91	0 (0–1)	3	3	100	4 (4–4)	88	68	77
5	16 (15/17)	63	56	89	18 (17–18)	70	65	93	2 (2–4)	10	2	20	2 (1–2)	6	6	100
6	18 (13–19)	874	750	86	52 (29–54)	2567	2258	88	1 (0–5)	70	66	94	3 (2–3)	147	92	63
7	7 (6–11)	98	83	85	21 (19–22)	268	245	91	0 (0–3)	12	12	100	4 (3–4)	51	38	75
8	22 (20–22)	129	110	85	8 (7–8)	46	43	93	0 (0–1)	2	1	50	3 (3–3)	18	17	94
9	16 (14–17)	754	674	89	15 (15–15)	705	701	99	0 (0–2)	18	18	100	3 (3–3)	138	137	99
10	17 (9–21)	859	731	85	15 (13–16)	724	652	90	0 (0–3)	14	14	100	1 (1–2)	51	49	96
All trials		2914	2529	87		5174	4691	91		136	123	90		522	421	81

*Note:* Not met criteria assessed for all trials; met, action needed, modifiable, and consent criteria assessed only for CTM eligible trials. All patients classified not eligible for trial 3.

*Abbreviations:* CTM: clinical trial matching; pt: patient.

In general, false positives and false negatives were the result of incorrect interpretation of eligibility criteria (IF THEN logic) by CTM. The most common cause of false positives and false negatives was misclassification of metastatic status in the context of progressive disease (ie, non-metastatic primary staging but metastatic at trial screening), summarized in Table 4.

**Table 4.** CTM misclassifications

Misclassification category	Misclassifications detail	Number of misclassifications per trial										
		1	2	3	4	5	6	7	8	9	10	All
Stage	Failure to detect metastatic disease status for patient with non-metastatic primary staging but metastatic disease at screening	0	3	0	1	1	3	0	1	3	2	14
	Failure to detect disease stage as not advanced/metastatic	0	0	0	2	0	4	3	0	2	6	17
	Failure to detect absence of measurable disease	0	0	0	4	0	0	0	0	0	0	4
	Detection of metastatic disease but failure to recognize as exclusion	0	0	0	0	0	13	0	0	0	0	13
Diagnosis	Failure to recognize mutation status as exclusion criteria	0	3	0	0	0	0	0	0	0	0	3
	Failure to recognize histology as exclusion criteria	0	0	0	0	0	1	0	0	0	0	1
Treatment	Failure to consider prior therapy as part of exclusion criteria	0	0	0	0	0	0	1	2	2	0	5
	Failure to consider timing of prior therapy as part of exclusion criteria	0	1	0	0	0	0	0	0	0	0	1
	Failure to consider location of prior radiotherapy as part of exclusion criteria	0	0	0	0	0	0	18	0	0	0	18
Pathology	Failure to apply correct logic relating to LFT in context of liver metastasis	0	0	0	0	2	0	0	0	0	0	2
Other	Exclusion rules for sub-cohorts applied to overall study	0	0	0	0	0	1	0	0	0	0	1
	Failure to recognize absence of progressive/recurrent disease	0	0	0	0	0	0	0	0	0	3	3

*Abbreviations:* CTM: clinical trial matching; LFT: liver function tests.

## DISCUSSION

This study is the first to evaluate performance of CTM eligibility determinations outside of the United States. In our unselected patient cohort, CTM software was able to reliably exclude ineligible patients from trial consideration (>95% accuracy), but less accurately identified eligible patients (77%). One contributing factor that limited CTM’s ability to determine eligibility was that for the 102-patient cohort, 8088 data items were identified as requiring further action (data input or clinician interpretation of eligibility). In routine use of CTM in clinical practice, this reconciliation process is part of the normal workflow, but it was not done as part of this retrospective study.

CTM performance in this cohort exceeded that of a previous study of CTM undertaken at Mayo Clinic in the United States in which accuracy was reported as 87.6% for 4 breast and 74.9% for 3 lung cancer trials.^12^ In the Mayo study, CTM used NLP to process unstructured electronic health record (EHR) documents to ingest patient data, whereas in this study, clinical data were entered into CTM directly. Both manual entry and NLP processes can introduce errors. While intended clinical utilization of CTM includes ingestion of patient data from an EHR, the hospital in our study did not yet have an integrated EHR. Therefore, our study tested CTM’s decision-making algorithms for trial eligibility, rather than its NLP capabilities in the patient ingestion process.

Our study evaluated combined NLP and ML performance of CTM for evaluation of eligibility criteria but did not separately evaluate these components. Zhang et al^13^ have reported on NLP classification methods for eligibility of HIV-positive patients for interventional cancer trials and eligibility of HIV-positive and pregnant women for general interventional trials. F2 scores (weighted average of precision and recall) ranged from 77% to 91% for these methods. Relative to a comparative trial matching platform from the Cincinnati Children’s Hospital Medical Center (CCHMC), CTM demonstrated significantly greater precision but lower recall and NPV. CCHMC developed and implemented its own clinical trial eligibility screening algorithm, reporting outcomes on 55 trial protocols and 215 pediatric oncology patients.^8^ Employing similar methods as used in our current study, CCHMC oncologists conducted manual medical record review for a randomly selected patient subset to generate a gold standard for performance assessment. In the CCHMC study, the best reported performance for matching trials to patients was 36% precision (vs 77% in the current study), 100% recall (vs 83%), 100% NPV (vs 96%), and 95.5% specificity (vs 94%). There are open-source tools to help with the process of clinical trials matching, however, the need for labeled data for NLP training and large datasets for ML can create obstacles to the success of open-source tools, many of which are developed in academic settings.^14^

Integration of clinical trial alert systems with EHRs has shown the potential to increase overall enrollment in trials, despite the alert fatigue noted by these studies.^15–18^

Tools such as Deep 6 AI,^19^ Mendel.ai,^20^ Antidote,^21^ Smart Patients,^22^ and Synergy^23^ are examples of artificial intelligence trial matching systems using ML and NLP. However, to the best of our knowledge there are no studies directly comparing these tools to each other. Publications in this area are mostly abstracts using limited datasets. Mendel.ai have published a retrospective study assessing the ability of their software to increase identification of eligible patients for 3 studies.^24^ For 2 of the studies, 24% and 50% potentially eligible patients were additionally identified. By comparison, our work analyzed a significantly larger number of studies, including phases I–III trials across a variety of treatment settings and modalities as available on ClinicalTrials.gov and recruiting at our institution.

We demonstrate the feasibility of developing and implementing an automated patient-trial classification system and highlight the clinical need for such systems, given the increasing challenge of manual matching and the trend toward increasingly complex, risk-based eligibility criteria that are not necessarily clinically intuitive. It is also the first such report using real-world data at an Australian cancer hospital. Importantly, we highlight the need for clinician input and oversight to support automated systems and remind the informatics community of the technical, intuitive, and nuanced clinician interpretations and decisions required to fully assess trial eligibility for an individual patient. CTM was designed and intended to be used as a clinical decision support tool to aid rather than replace clinicians in determining trial eligibility.

The current study has several limitations. First, although CTM is capable of processing structured and unstructured information from an EHR, only the matching components (NLP and ML performance) of the CTM system were evaluated, because an EHR was not integrated with CTM for this study. Second, we did not separately evaluate the NLP and ML performance of CTM for eligibility assessment. Third, the study included a relatively small number of patients at a single center. Fourth, though reduction in manual labor is a benefit of automated systems such as CTM, the manual entry of data in this study (necessitated by the lack of an integrated EHR) did not accurately reflect standard processes and so is not likely to be representative of results using automated systems.

Strengths of the study are a rigorous gold standard for eligibility with consensus agreement of 2 clinicians with high interrater reliability. We also recognize that the clinician-derived gold standard in this study is not feasible for larger scale studies. For larger studies, it is likely that automated or semi-automated EHR data extraction would be required, though such methods have their own limitations. Therefore, we highlight the high value in a small dataset that has had human review of every case as a strength of this study.

## CONCLUSION

This study demonstrated that CTM allows efficient and reliable screening of Australian lung cancer patients for clinical trials, with 96% accuracy in exclusion and 92% performance in assessing overall potential eligibility. Many patient attributes remained unknown after CTM analysis, highlighting the need for clinician input and oversight in assessing nuances of patient characteristics against individual criteria.

## FUNDING

This work was funded by IBM.

## AUTHOR CONTRIBUTIONS

DMH initiated/conceptualized the manuscript and collected data for the manuscript. MA drafted the manuscript and analyzed the data. All authors contributed to the interpretation of data, critically revised the manuscript, and approved the final version.

## SUPPLEMENTARY MATERIAL

Supplementary material is available at *Journal of the American Medical Informatics Association* online.

## Supplementary Material

ooaa002_Supplementary_Data

## Notes

This work was presented, in part, as a podium (oral) presentation at the 2019 AMIA Annual Symposium, Washington, D.C., 19 November, 2019.
