# Supplementary material for: Evaluation of an artificial intelligence clinical trial matching system in Australian lung cancer patients
Source: JAMIA Open. 2020 May 1;3(2):209–15. doi: 10.1093/jamiaopen/ooaa002 (PMC7382632; doi:10.1093/jamiaopen/ooaa002)
Supplement: ooaa002_Supplementary_Data [file ooaa002_supplementary_data.docx]

**Supplementary Table 1**. Clinical trial features

| **Trial No** | **Phase** | **Design** | **Intervention** | **Population** |
| --- | --- | --- | --- | --- |
| 1 | 3 | 2 arm RCT | adjuvant ALK inhibitor versus adjuvant platinum-based chemotherapy | Resected stage IB-III ALK positive NSCLC |
| 2 | 2 | Single arm | Study drug plus Pembrolizumab | Advanced or metastatic solid tumors |
| 3 | 3 | 2 arm RCT | Neoadjuvant platinum-based chemotherapy plus PD-L1 antibody or placebo | Resectable stage IB-IIIA NSCLC |
| 4 | 1b | Single arm | Combination oral therapy | Advanced or metastatic KRAS/BRAF mutant NSCLC |
| 5 | 3 | 2 arm RCT | Comparison of ALK inhibitors | Treatment naive ALK positive NSCLC |
| 6 | 1b/2 | Multi arm umbrella study | Multiple immunotherapy-based treatment combinations | Metastatic NSCLC |
| 7 | 1 | 2 arm RCT | PD-1 antibody before or after Stereotactic Ablative Body Radiotherapy | Metastatic NSCLC |
| 8 | 3 | 2 arm RCT | Adjuvant PD-L1 antibody versus placebo | Resected NSCLC |
| 9 | 1 | Single arm | oral C-MET selective Tyrosine Kinase Inhibitor | advanced cancer |
| 10 | 1 | Single arm | RET inhibitor | RET rearranged thyroid cancer and NSCLC |

**Abbreviations**: ALK - anaplastic lymphoma kinase; NSCLC – non small cell lung cancer; RCT – randomized controlled trial, PD-1 – programmed death-1, PD-L1 programmed death ligand-1
